# Age-Related Differences in Spatial Frequency Processing during Scene Categorization

**DOI:** 10.1371/journal.pone.0134554

**Published:** 2015-08-19

**Authors:** Stephen Ramanoël, Louise Kauffmann, Emilie Cousin, Michel Dojat, Carole Peyrin

**Affiliations:** 1 Univ. Grenoble Alpes, LPNC, F-38000 Grenoble, France; 2 CNRS, LPNC, F-38000 Grenoble, France; 3 Univ. Grenoble Alpes, GIN, F-38000 Grenoble, France; 4 UMS IRMaGe, F-38000 Grenoble, France; 5 INSERM U836, GIN, F-38000 Grenoble, France; University Medical Center Goettingen, GERMANY

## Abstract

Visual analysis of real-life scenes starts with the parallel extraction of different visual elementary features at different spatial frequencies. The global shape of the scene is mainly contained in low spatial frequencies (LSF), and the edges and borders of objects are mainly contained in high spatial frequencies (HSF). The present fMRI study investigates the effect of age on the spatial frequency processing in scenes. Young and elderly participants performed a categorization task (indoor vs. outdoor) on LSF and HSF scenes. Behavioral results revealed performance degradation for elderly participants only when categorizing HSF scenes. At the cortical level, young participants exhibited retinotopic organization of spatial frequency processing, characterized by medial activation in the anterior part of the occipital lobe for LSF scenes (compared to HSF), and the lateral activation in the posterior part of the occipital lobe for HSF scenes (compared to LSF). Elderly participants showed activation only in the anterior part of the occipital lobe for LSF scenes (compared to HSF), but not significant activation for HSF (compared to LSF). Furthermore, a ROI analysis revealed that the parahippocampal place area, a scene-selective region, was less activated for HSF than LSF for elderly participants only. Comparison between groups revealed greater activation of the right inferior occipital gyrus in young participants than in elderly participants for HSF. Activation of temporo-parietal regions was greater in elderly participants irrespective of spatial frequencies. The present findings indicate a specific low-contrasted HSF deficit for normal elderly people, in association with an occipito-temporal cortex dysfunction, and a functional reorganization of the categorization of filtered scenes.

## Introduction

Our visual world is hierarchically organized. Visual scenes of daily life, such as outdoor or indoor environments, contain many visual objects made of smaller objects comprising multiple visual details. There is considerable evidence suggesting that the spatial frequency content of scenes is important for organized perception. Convergent data from the functional neuroanatomy of magnocellular and parvocellular visual pathways [1], neurophysiological recordings in primates (for a review, see [2]), psychophysical studies in humans [3,4], and simulations [5,6] suggest that the first stage of visual perception consists of the parallel extraction of different visual elementary features at different spatial frequencies. Low spatial frequencies (LSF), conveyed by fast magnocellular pathways, provide coarse information about the scene (e.g., the global shape and structure), whereas high spatial frequencies (HSF), conveyed more slowly by the parvocellular pathways, provide more detailed information about the scene (e.g., the edges and borders of an object). Temporal precedence of LSF over HSF processing has generally been observed in behavioral studies using sinusoidal grating [3,4,7] and complex filtered scenes as stimuli [8,9,10].

Recent neuroimaging studies have aimed to identify the cerebral regions differentially involved in LSF and HSF processing in young adults (for a recent review, see [11]). Using either sinusoidal gratings or more complex scene stimuli, recent studies revealed that spatial frequency processing is retinotopically organized in the visual cortex [12,13,14]. More precisely, LSF scene categorization activated occipital areas in relation to the peripheral representation of the visual field, whereas HSF scene categorization activated occipital areas in relation to the foveal representation. However, the way spatial frequencies are processed in normal aging remains unclear.

Normal aging is characterized by a decline in many cognitive functions, such as selective attention [15,16,17], working memory [18], processing speed [19,20] and executive control [21]. Many visual functions, such as visual acuity [22,23,24,25] and contrast sensitivity [26,27,28,29,30] also decline with age. Studies on spatial contrast sensitivity present conflicting results, but the majority have shown that, with increasing age, contrast sensitivity decreases mainly for medium and high spatial frequencies [26,27,29,30]; see however [31]. Owsley, Sekuler and Siemsen [30] showed that contrast sensitivity for stationary LSF gratings remained the same throughout adulthood, whereas sensitivity for stationary HSF gratings decreased with age (beginning at around 40 to 50 years), suggesting more pronounced impairment of the parvocellular pathway. Hardy et al. [32] measured sensitivity to chromatic contrasts (e.g., green and red contrast) for different spatial frequencies using sinusoidal gratings in young and older participants. Results showed that the threshold of sensitivity to chromatic contrast was higher in older than in younger participants, especially for HSF gratings. Given the parvocellular pathway’s sensitivity to chrominance and HSF, these results have been interpreted in favor of a functional decline of the parvocellular pathway in normal aging. Elliot and Werner [33] directly investigated age-related changes in magno- and parvocellular pathways using two paradigms thought to separate these two pathways based on their contrast gain signature [34]. Results showed a functional deficit of the two pathways with age, and this was more pronounced in the parvocellular pathway. Loss of contrast sensitivity for HSF with increasing age could, therefore, be explained by a higher sensitivity of the parvocellular pathway to normal aging.

Similarly, behavioral experiments investigating the perception of global and local visual information in normal elderly people have produced conflicting results on the processing of spatial frequencies. Studies using hierarchical stimuli (global forms composed of several local elements; [35]) show that young adults identify the global form more rapidly than local elements (global precedence effect). Based on the assumption that global information is preferentially conveyed by LSF, and that local information is conveyed by HSF [36,37,38], the global precedence effect has been interpreted as additional evidence of the temporal precedence of LSF over HSF processing. However, a number of studies have shown that with age, the advantage of global processing tends to be reversed in favor of local processing [39,40,41]. Lux et al. [39] for example reported that reaction times of young adult participants were faster when detecting global forms, while older participants had faster reaction times when detecting local elements. These results could be interpreted as reflecting a temporal precedence of HSF over LSF processing in normal elderly people. However, in other studies, global precedence was not reduced, and became even more pronounced with increased age [42,43].

Only a few studies have investigated the effects of age on spatial frequency processing using more complex and ecological stimuli than gratings and hierarchical forms. Viggiano, Righi and Galli [44] presented sequences of nine images of objects (animals or tools). Sequences started with an LSF filtered object and HSF information was added progressively. Participants had to identify the object in each image in these sequences. Results showed that in order to identify the objects correctly, older participants needed more HSF information than young participants. Musel et al. [8] presented sequences of six images of scenes (indoors or outdoors) in which the spatial frequency content differed from one image to the other, going either from LSF to HSF or from HSF to LSF. Results showed that young participants categorized low-to-high sequences more quickly than high-to-low sequences, consistent with the temporal advantage of LSF over HSF. The LSF advantage tended to be reversed in older participants (over the age of 60). These two studies suggest that HSF are more important to older than to younger participants for visual perception. However, because it was not possible to compare HSF and LSF directly, the authors were unable to reach any conclusion on the existence of a potential deficit in spatial frequency processing related to normal aging. Musel et al. [45] tested the categorization of LSF and HSF scenes in normal elderly participants in order to establish normative data to assess the processing of spatial frequencies in age-related macular degeneration. Results showed that the performance of healthy elderly participants was not affected by the spatial frequency content of scenes, but provided no answer concerning a possible deficit in spatial frequency processing related to normal aging because no comparison with young participants was made. Overall, these behavioral studies suggest that the visual mechanisms involved in spatial frequency processing may change with age.

The small number of neuroimaging studies which have investigated age-related differences on visual processing reveal cortical anatomical changes, characterized by atrophy of white and gray matter in the primary visual cortex with age [46,47], and changes in the retinotopy of visual areas [48,49,50]. The latter may influence the way spatial frequencies are processed in the occipital cortex. However to our knowledge, no studies have as yet been conducted on the effects of age on the neural bases of spatial frequency processing.

The present study was adapted from a previous fMRI study conducted in young adults only [13] to investigate elderly adults’ ability to process spatial frequencies in natural environments compared to that of young adults. For this purpose, young and elderly participants had to categorize natural indoor and outdoor scenes filtered in LSF and HSF. Exemplars from the outdoor and indoor categories were chosen in order to have similar dominant orientations in the amplitude spectrum and to avoid categorization based on this type of visual cue. From a pragmatic point of view, this categorization task can be performed whatever the type of filtering (low-pass, high-pass, or pass-band). Furthermore, this task is simple and quick to administer, but also easy to perform even for patients with age-related degeneration (Musel et al., 2011). It should be noted that luminance contrast in scenes decreases as spatial frequency increases, following a 1/fα function [51]. Therefore, LSF scenes are characterized by high luminance contrast, while HSF scenes are characterized by low luminance contrast. FMRI was used to investigate any neural correlates of spatial frequency changes due to age-related differences in the whole brain. Furthermore, most studies agree that the parahippocampal cortex, especially the posterior part known as the parahippocampal place area (PPA), is a region of the human cortex involved in the processing of visual scenes. Early studies [52,53,54,55,56], showed that the PPA responds more strongly to images of real-world scenes (such as cityscapes and landscapes) than to other meaningful visual stimuli (such as faces and objects). We also investigated age-related differences in spatial frequency processing in the PPA. Studying the effects of normal aging on the structural and functional properties of cortex in human is essential to distinguish cortical changes related to healthy aging from those resulting from the pathophysiology of an underlying disease [49].

## Methods

### Participants

Twenty-four right-handed participants divided into two age groups were included in the experiment (Table 1): 12 young participants (6 males; Mean age ± SD: 22 ± 3 years; Range 18–26 years) and 12 elderly participants (8 males; Mean age ± SD: 64 ± 3 years; Range 61–71 years) with normal or corrected-to-normal vision. Visual acuity was tested prior to the experiment, using the Monoyer chart designed for testing long distance vision. Participants requiring visual correction wore the MediGoggle Adult Research Set (Cambridge Research Systems Ltd, England; http://www.crsltd.com/), interchangeable prescriptive goggles suitable for use in MR environments. Participants had no neurological or ocular disorders (such as age-related macular degeneration, glaucoma or multiple sclerosis). All participants gave their informed written consent before participating in the study, which was carried out in accordance with The Code of Ethics of the World Medical Association (Declaration of Helsinki) for experiments involving humans, and approved by the local ethics committee (Comité de protection des personnes Sud-Est V, ID RCB: 2011-A01551-40).

**Table 1:** Age and visual acuity of Young (Y) and Elderly (E) participants.

Participants	Age	Visual Acuity		Participants	Age	Visual Acuity	
	(years)	(Log MAR)			(years)	(Log MAR)	
		left eye right eye				left eye right eye	
Y1	22	0.0	0.0	E1	60	0.0	0.0
Y2	25	0.0	0.0	E2	61	0.3	0.2
Y3	24	-0.1	-0.1	E3	71	0.5	0.0
Y4	18	0.0	0.0	E4	66	0.0	0.0
Y5	18	-0.1	-0.1	E5	62	0.0	0.0
Y6	25	-0.1	0.2	E6	61	0.3	0.2
Y7	23	-0.1	0.0	E7	64	0.0	0.0
Y8	24	0.0	0.0	E8	61	0.2	0.2
Y9	18	0.0	0.0	E9	67	0.3	0.0
Y10	23	0.0	0.0	E10	64	-0.1	-0.1
Y11	25	0.0	0.0	E11	68	0.0	0.0
Y12	19	0.1	0.2	E12	65	0.2	0.2

### Stimuli and Procedure in the Spatial Frequency Experiment

Stimuli consisted of 20 black and white photographs (256-level grey-scales, 1042x768 pixels) of scenes classified into two distinct categories (10 indoor scenes and 10 outdoor scenes) with a visual angle of 24x18 degrees, ensuring to stimulate both the fovea and the peripheral visual field [13]. Outdoor scenes are views of houses or buildings with sky at the top and outdoor-relevant objects (e.g., car, tree). Indoor scenes are kitchens, offices and living rooms with indoor-relevant objects (e.g., table, sofa, chair). Scenes were displayed in their original version and in their mirrored version (left and right were reversed) in order to avoid any effect of the visual asymmetry of these large scene images. Exemplars from the two categories (outdoor and indoor) were chosen in order to have similar dominant orientations in the amplitude spectrum and to avoid categorization based on this type of visual cue.

To ensure that the chosen scenes have similar amplitude spectra, we first calculated the mean amplitude spectrum for the 10 indoor scenes (mean AS indoor) and the 10 outdoor scenes (mean AS outdoor). Then, for each scene, we calculated two 2D correlation coefficients, one between the sceneʼs amplitude spectrum and the mean AS indoor and the other one between the sceneʼs amplitude spectrum and the mean AS outdoor. The mean AS of the category corresponding to the scene of interest was calculated by excluding the sceneʼs amplitude spectrum (i.e., for an indoor scene, the mean AS indoor was calculated based on the 9 remaining indoor scenes, whereas the mean AS outdoor was calculated based on the 10 outdoor scenes). The 2D correlation coefficient was calculated using the Matlab function “corr2d.” A 2 × 2 variance analyses (ANOVA) with the category of the scene (indoor and outdoor) and the category of the mean AS (indoor and outdoor) as within-subject factors were conducted on the 2D correlation coefficients. Results show that the 2D correlation coefficients calculated between indoor scenes and the mean AS indoor did not significantly differ from those calculated between indoor scenes and the mean AS outdoor (0.76 ± 0.05 and 0.76 ± 0.05, respectively; F_1,18_ < 1). Similarly, the 2D correlation coefficients calculated between outdoor scenes and the mean AS outdoor did not significantly differ from those calculated between outdoor scenes and the mean AS indoor (0.78 ± 0.05 and 0.78 ± 0.04, respectively; F_1,18_ < 1). Outdoor and indoor categories were equivalent in terms of visual cluttering (Subband Entropy measures; see [57]). Mean subband entropy was equivalent for outdoors and indoors (2.91 ± 0.15 and 2.91 ± 0.15, respectively; F_1,18_ < 1).

**Fig 1:**
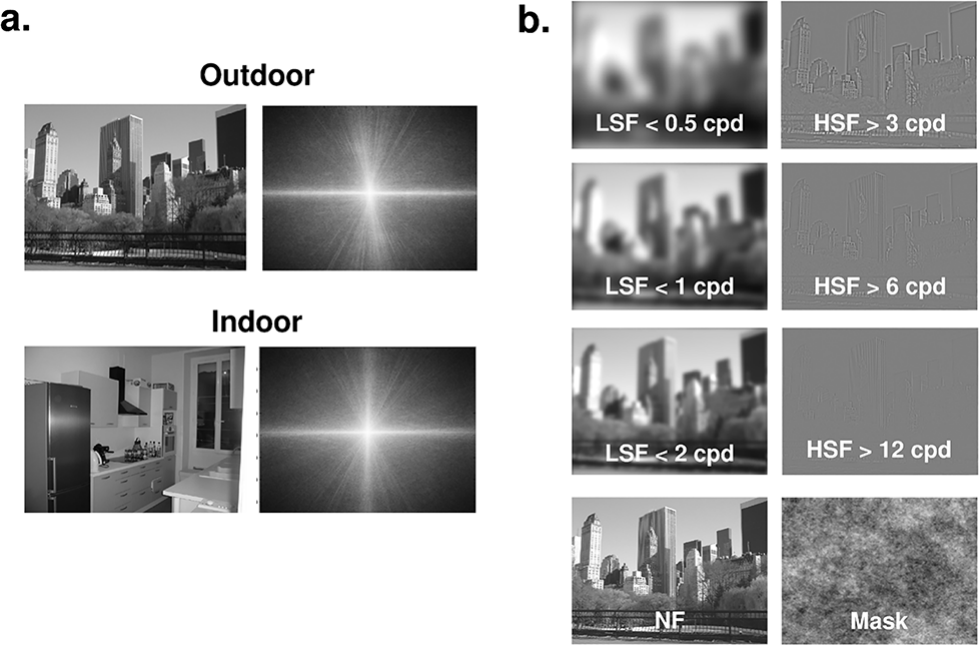
(a) Examples of scenes belonging to two different categories (outdoors and indoors) with the mean amplitude spectrum of each category. On the amplitude spectrum images, low spatial frequencies are close to the center and high spatial frequencies are on the periphery. Vertical orientations are represented on the x-axis and horizontal orientations on the y-axis. (b) Example of non-filtered scenes (NF), low-spatial frequency scenes (LSF) below 0.5, 1, and 2 cycles per degree (cpd), and high-spatial frequency scenes (HSF) above 3, 6, and 12 cpd. The mask used is also presented (bottom). It should be noted that the perception of spatial frequencies could be affected by picture reduction of scenes for illustrative purposes. In the experiment, picture size was about 10 times bigger than in this figure.

Stimuli were elaborated using the image processing toolbox on MATLAB (Mathworks Inc., Sherborn, MA, USA). Each scene was filtered with three low-pass filters, with cutoff frequencies corresponding to 0.5, 1, and 2 cycles per degree (cpd; i.e. 12, 24, 49 cycles per image) and three high-pass filters with cutoff frequencies corresponding to 3, 6, and 12 cpd (i.e. 71, 144, 293 cycles per image), or left unfiltered (NF). Cut-off frequencies followed a logarithmic scale in order to obtain a better sampling of the amplitude spectrum of natural scenes (see [58] for a similar procedure). Furthermore, these values were chosen in order to include cut-off frequencies of 2 cpd for LSF and 6 cpd for HSF, as used in Schyns & Oliva’s pioneer study on spatial frequency processing during scene perception [10]. The resulting images were then normalized to obtain a mean luminance equal to 128 on a grey-level scale ranging from 0 to 256. This resulted in 7 versions of each scene (1 NF, 3 LSF and 3 HSF, see Fig 1). Stimuli were displayed using E-prime software (E-prime Psychology Software Tools Inc., Pittsburgh, USA) and back-projected onto a translucent screen positioned at the rear of the magnet. Participants viewed this screen at a distance of about 222 cm via a mirror fixed on the head coil. We used a backward mask, built with 1/f white noise, to prevent retinal persistence of the scene.

We used a block-design paradigm with the NF, LSF, and HSF scenes. The Spatial Frequency experiment consisted of four functional runs. Each functional scan lasted five minutes and was composed of nine 25-second task blocks (one block per spatial frequency cut-off and three blocks of NF scenes), including 10 scenes (5 indoors and 5 outdoors), interspersed with three 25-second blocks with a fixation dot in the center of the screen (Fixation condition) displayed against a grey background. Each scene was presented in all spatial frequency conditions. The order of images was randomized within blocks. It should be noted that the block-design paradigm did not allow us to analyze individual responses to trials in the way an event-related paradigm would have, and it did not allow us to investigate the neural correlates of semantic category effect and priming effect in our study. Each stimulus was displayed for 100 ms, followed by a mask for 30 ms and a fixation dot in the center of the screen. The interval between the onsets of two successive stimuli was 2.5 seconds. Participants had to give a categorical answer on the scenes (“indoors” or “outdoors”) by pressing the corresponding key with the forefinger and the middle finger of their dominant hand. They were instructed to fixate on the center of the screen (fixation dot) during the entire run and to respond as quickly and as accurately as possible by pressing one of two response buttons. Half of the participants had to answer “indoors” with their forefinger and “outdoors” with their middle finger, while the other half had to answer ‘indoors” with their middle finger and “outdoors” with their forefinger. Response accuracy and reaction times (RT, in milliseconds) were recorded. If participants were not able to categorize the scene, they were allowed to not give a response. An error could be either a non-response or a false categorization. Analyses were conducted on mean non-response error rate (mNR), mean false categorization rate (mFC) and mean correct reaction times (mRT).

### Stimuli and Procedure in the Localizer experiment of scene-selective areas

Following the main Spatial Frequency experiment, we performed a separate functional Localizer experiment (adapted from [59,60]) to localize the PPA. Participants viewed grayscale photographs of scenes, faces, and common objects in separate blocks of a block design paradigm. Scene pictures used in the localizer experiment were not shown during the spatial frequency experiment. Stimuli were black and white photographs (256 greyscales), all sized 700 x 700 pixels (or 16.4 x 16.4 degrees of visual angle). The localizer experiment consisted of one functional run. The functional run lasted 3 minutes and was composed of eight 15-s task blocks (four blocks of scenes, two blocks of faces, and two blocks of objects), including 15 different images of the same type, interspersed with four 15-s blocks with a fixation dot in the center of the screen displayed against a gray background. Participants performed a “one-back” repetition detection task. They were instructed to press a button whenever they saw two identical stimuli repeated. Only two repetitions per block were presented. Each stimulus was presented for 300 ms, with a 700 ms interstimulus interval with a fixation dot in the center of the screen. For each participant, the PPA was identified in both hemispheres by a [Scenes > Faces + Objects] contrast.

### fMRI Acquisition

Experiments were performed using a whole-body 3T Philips scanner (Achieva 3.0T TX Philips—Philips Medical Systems, Best, NL) with a 32-channel head coil at the Grenoble MRI facility IRMaGe in France. For all functional Spatial Frequency scans, the manufacturer-provided gradient-echo/T2* weighted EPI method was used. Forty-four adjacent axial slices parallel to the bi-commissural plane were acquired in sequential mode. Slice thickness was 3 mm. The in-plane voxel size was 2.5×2.5 mm (220×220 mm field of view acquired with a 88×85 pixel data matrix; reconstructed with zero filling to 96×96 pixels). The main sequence parameters were: TR = 2.5 s, TE = 30 ms, flip angle = 80°. Finally, a T1-weighted high-resolution three-dimensional anatomical volume was acquired, by using a 3D Modified Driven Equilibrium Fourier Transform (MDEFT) sequence (field of view = 256×224×175 mm; resolution: 1.33×1.70s×1.37 mm; acquisition matrix: 192×132×128 pixels; reconstruction matrix: 288×288×128 pixels).

### Data Analysis

Data analysis was performed using the general linear model [61] for block designs in SPM12b (Wellcome Department of Imaging Neuroscience, London, UK, www.fil.ion.ucl.ac.uk/spm/) implemented in MATLAB 7 (Mathworks Inc., Sherborn, MA, USA). Functional volumes were realigned to correct for head movements to the mean functional image using a rigid body transformation. The T1-weighted anatomical volume was then realigned (affine transformation) to match the mean functional image of each participant, and was then normalized (non rigid, non linear transformation) into the MNI space. A default 4th degree B-Spline interpolation was applied. The anatomical normalization parameters were subsequently used for the normalization of functional volumes. Finally, each functional volume was smoothed by an 8-mm FWHM (Full Width at Half Maximum) Gaussian kernel. Times-series for each voxel were high-pass filtered (1/128 Hz cutoff) to remove low-frequency noise and signal drift. We used an efficient non-linear deformation algorithm [62] with a high level number of degrees of freedom to realign all individual brains, and by visual inspection, we did not notice any realignment problem in some parts of the brain between elderly and young anatomical scans.

For the Spatial Frequency experiment, eight conditions of interest (LSF-0.5cpd, LSF-1cpd, LSF-2cpd, HSF-3cpd, HSF-6cpd, HSF-12cpd, NF, and Fixation) were modeled as eight regressors constructed as box-car functions convolved with a canonical hemodynamic response function for each participant. Accuracy for each trial (either correct responses or error) and movement parameters derived from realignment corrections (three translations and three rotations) were also entered in the design matrix as additional factors of no interest to account for no-response related variance and head motion, respectively. On an individual level, we first identified the brain regions involved in the processing of each spatial frequency content relative to the fixation ([LSF > fixation], [HSF > fixation], and [NF > fixation]), irrespective of the spatial frequency cut-off. We also tested the effect of spatial frequency cut-off for each spatial frequency band relative to the fixation ([LSF-05cpd > fixation], [LSF-1cpd > fixation], [LSF-2cpd > fixation], [HSF-3cpd > fixation], [HSF-6cpd > fixation], [HSF-12cpd > fixation]). To allow population inference, three ANOVAs were performed based on individual analysis by means of a flexible-factorial design following the guidelines by Glascher and Gitelman [63]. The first ANOVA aimed to test the interaction between groups and the processing of spatial frequencies. We modeled groups (young and aged) and spatial frequency bands (LSF, HSF, and NF) as factors. This ANOVA allowed us to investigate the processing of spatial frequencies in each group separately, and to test differences between young and aged participants on each spatial frequency band. The second ANOVA aimed to assess the effect of LSF cut-off on within- and between-group differences. We modeled groups (young and aged) and LSF cut-offs (LSF-0.5cpd, LSF-1cpd, and LSF-2cpd) as factors. The third ANOVA aimed to assess the effect of HSF cut-off on within- and between-group differences. We modeled groups (young and aged) and HSF cut-offs (HSF-3cpd, HSF-6cpd, and HSF-12cpd) as factors. Areas of activation were considered significant if they exceeded a voxel and cluster threshold of p < 0.05 family-wise error (FWE) corrected for multiple comparisons, with a minimum cluster extent of 5 voxels (T < 5.18 for the first ANOVA and T < 5.23 for the second and third ANOVA). To facilitate comparisons with other studies, a transformation of MNI into Talairach and Tournoux [64] coordinates was performed using the MNI2TAL function (created by Matthew Brett, available at www.mrc-cbu.cam.ac.uk/Imaging).

For the Localizer experiment, the fMRI signal in the localizer run was analyzed using single-participant general linear model. For each participant, four conditions of interest (scenes, faces, objects, and fixation) were modeled as four regressors, constructed as box-car functions convolved with a canonical hemodynamic response function. Movement parameters derived from realignment corrections (three translations and three rotations) were also entered into the design matric as additional factors of no interest. Left and right PPA were defined independently for each participant as regions of interest (ROIs) using the [Scenes > Faces + Objects] contrast. Significant voxel clusters on individual t maps were identified using a false-discovery correction at qFDR < .05 to control for the overall false-positive rate. As the clusters were generally large and involved several regions of interest, small sphere ROIs (3 mm radius) were created at individual peaks of activation in each scene-selective region and in each hemisphere. These clusters were selected as ROIs for the data analysis in the Spatial Frequency experiment. Parameter estimates (% signal change relative to the global mean intensity of signal) of block responses were extracted from the two sphere ROIs for each participant. The average parameter of activity was calculated for the NF, LSF, and HSF. These values were submitted to an ANOVA for mixed designs with Group (Young and Elderly) as between-subject factor, and Hemisphere (Left and Right) and Spatial Frequency (NF, LSF, and HSF) as within-subject factors. In order to examine whether activity in PPA was influenced by the spatial frequency cut-offs for the LSF and HSF scenes, we additionally performed two separate ANOVAs for mixed designs with Groups (young and aged participants) as between-subject factor, and Hemisphere (Left and Right) and Spatial frequency cut-off (either 0.5, 1, and 2 cpd for LSF scenes or 3, 6, and 12 cpd for HSF scenes) as within-subject factors.

## Behavioral Results

Three 2x3x2 analyses of variance (ANOVA) with Group (young and elderly participants) as between-subjects factor, and Spatial frequency (NF, LSF, and HSF) and Category (indoors and outdoors) as within-subjects factors were conducted on mNR, mFC and mRT.

**Fig 2:**
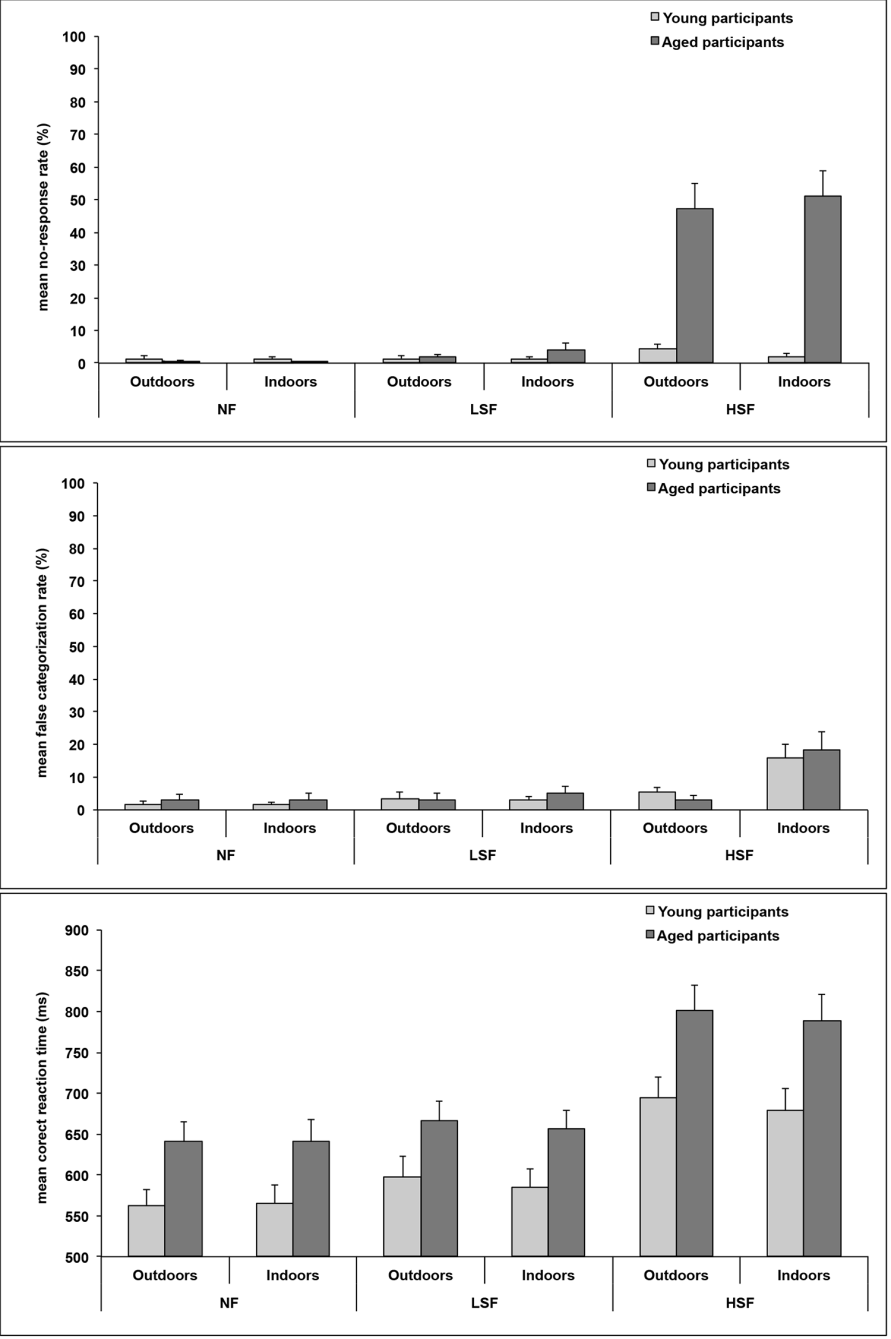
Mean no-response error rates (%), mean false categorization rates (%) and mean correct reaction times (in milliseconds) for the categorization of non-filtered scenes (NF), low-spatial frequency scenes (LSF), and high-spatial frequency scenes (HSF). Error bars correspond to the standard error.

The ANOVA on mNR (Fig 2) revealed that elderly participants responded less often than young participants (Mean ± SD: 17.5 ± 10.9% and 1.9 ± 3.4%, respectively; F_1,22_ = 26.54, p < 0.001). The expected Group x Spatial frequency interaction was significant (F_2,44_ = 37.35, p < 0.001). Planned comparison showed that elderly participants responded less often when categorizing HSF scenes (49.2 ± 26.9%) than NF scenes (0.3 ± 0.8%; F_1,22_ = 80.79, p < 0.001) and LSF scenes (2.8 ± 5.0%; F_1,22_ = 85.01, p < 0.001), and when categorizing LSF than NF scenes (F_1,22_ = 7.52, p < 0.05). For young participants, there was no effect of spatial frequencies (NF: 1.2 ± 3.6%; LSF: 1.3 ± 2.8%; HSF: 3.3 ± 3.90%; all F_1,22_ < 1). In addition, elderly participants responded less often than young participants only when categorizing HSF scenes (F_1,22_ = 34.67, p < 0.001; NF: F_1,22_ < 1; LSF; F_1,22_ = 1.01, p = 0.33). The Group x Spatial frequency x Category interaction was significant (F_2,44_ = 5.76, p < 0.01). Planned comparison revealed a significant Spatial frequency x Category interaction only for elderly participants (F_2,44_ = 4.77, p < 0.05; young participants: F_2,44_ = 1.80, p = 0.18) due to the fact that they only responded less often for categorizing indoor than outdoor scenes filtered in HSF (51.1 ± 27.2% and 47.4 ± 26.6%, respectively; F_1,22_ = 11.03, p < 0.01; NF: F_1,22_ = 1.11, p = 0.305; LSF: F_1,22_ = 3.13, p = 0.09).

The ANOVA on mFC (Fig 2) did not show a main effect of Groups (F_1,22_ < 1), interaction between Groups and Spatial frequencies (F_2,44_ < 1) or interaction between Groups, Spatial frequencies and Categories (F_2,44_ < 1). There was only a main effect of Spatial frequency (F_2,44_ = 18.23, p < 0.001). All participants made more false categorizations when categorizing HSF scenes (10.7 ± 10.9%) than NF scenes (2.4 ± 5.0%; F_1,22_ = 22.04, p < 0.001) and LSF scenes (3.7 ± 6.1%; F_1,22_ = 15.48, p < 0.001), and when categorizing LSF than NF scenes (F_1,22_ = 6.39, p < 0.05).

Finally, the ANOVA on mRT (Fig 2) revealed a main effect of Groups (F_1,22_ = 7.05, p < 0.05). Elderly participants were slower to categorize scenes than healthy participants (699 ± 92 and 614 ± 81 ms, respectively). The expected Group x Spatial frequency interaction was not significant (F_2,44_ = 1.43, p = 0.25), and the Group x Spatial frequency x Category interaction was not significant (F_2,44_ < 1). There was a main effect of Spatial frequency (F_2,44_ = 77.07, p < 0.001). All participants had longer RT when categorizing HSF scenes (741 ± 101 ms) than NF scenes (603 ± 79 ms; F_1,22_ = 86.90, p < 0.001) and LSF scenes (626 ± 81 ms; F_1,22_ = 69.83, p < 0.001), and when categorizing LSF than NF scenes (F_1,22_ = 31.65, p < 0.001).

The relationship between visual acuity and performance was statistically assessed for each spatial frequency condition by using Pearson correlation tests between each group’s performance (mNR, mFC, and mRT) and visual acuity. Results for young participants showed no correlations between LSF and Visual acuity (mNR: r = -0.19, p = 0.56; mFC: r = -0.05, p = 0.89; mRT: r = 0.29, p = 0.36) or HSF and Visual acuity (mNR: r = -0.26, p = 0.42; mFC: r = -0.01, p = 0.99; mRT: r = 0.17, p = 0.59). For elderly participants, there was a significant correlation between LSF and Visual acuity on the mFC (r = 0.60, p < 0.05). The lower participants’ visual acuity, the higher were their false categorization rates. However, descriptive analysis on single participants data showed that the mFC of the elderly participant with the worst visual acuity (E6 in Table 1) was the higher (26%), well above the group average (4 ± 7%), and suggest that the significant correlation could be due to this outlier. When removing this participant, results did not show significant correlation between LSF and Visual acuity (r = 0.24, p = 0.48). No correlation was observed between LSF and Visual acuity for the mNR (r = -0.23, p = 0.47) and the mRT (r = -0.09, p = 0.78) and between HSF and Visual acuity for either measure (mNR: r = 0.35, p = 0.26; mFC: r = -0.07, p = 0.82; mRT: r = 0.03, p = 0.92).

We also tested whether the spatial frequency cut-off for the LSF and HSF conditions could influence mNR. Separate ANOVAs were conducted for the LSF and HSF stimuli with Group (young and elderly participants) as between-subjects factor, and Spatial frequency cut-off (either 0.5, 1, and 2 cpd for LSF scenes or 3, 6, and 12 cpd for HSF scenes) and Category (indoors and outdoors) as within-subjects factors. For the LSF scenes, there was neither Group x Spatial frequency cut-off interaction (F_2,44_ = 2.53, p = 0.09), nor Group x Spatial frequency cut-off x Category interaction (F_2,44_ = 1.31, p = 0.28). There was only a main effect of Spatial frequency cut-off (F_2,44_ = 3.77, p < 0.05). However, planned comparisons did not show that participants responded less often as the cut-off for the LSF information decreased (0.5 cpd: 4.4 ± 8.5%; 1 cpd: 0.7 ± 2.0%; 2 cpd: 1.0 ± 2.5%; 0.5 vs. 1 cpd: F_1,22_ = 3.72, p = 0.07; 0.5 vs. 2 cpd: F_1,22_ = 4.07, p = 0.07; 1 vs. 2 cpd: F_1,22_ < 1). For the HSF scenes, there was a significant Group x Spatial frequency cut-off interaction (F_2,44_ = 27.74, p < 0.001). Planned comparisons revealed that elderly participants responded less often as the cut-off for the HSF information increased (3 cpd: 14.6 ± 20.5%; 6 cpd: 57.7 ± 35.4%; 12 cpd: 75.4 ± 35.3%; 3 vs. 6 cpd: F_1,22_ = 62.70, p < 0.001; 3 vs. 12 cpd: F_1,22_ = 81.38, p < 0.001; 6 vs. 12 cpd: F_1,22_ = 15.70, p < 0.001), but no effect of cut-off was observed for young participants (3 cpd: 2.1 ± 3.8%; 6 cpd: 1.3 ± 2.4%; 12 cpd: 6.5 ± 8.7%; all F_1,22_ < 1). In addition, elderly participants responded less often than young participants for all spatial frequency cut-off of HSF scenes (3 cpd: F_1,22_ = 4.43, p < 0.05; 6 cpd: F_1,22_ = 31.05, p < 0.001; 12 cpd: F_1,22_ = 44.88, p < 0.001). The Group x Spatial frequency cut-off x Category interaction was not significant (F_1,44_ < 1).

It should be noted that luminance contrast is higher for LSF than for HSF. A control behavioral study was conducted in the same participants to verify that the specific HSF deficit in elderly participants did not result from a lower contrast in HSF scenes (see “Supplementary Material”). We have equalized the luminance contrast of filtered stimuli using the RMS (root mean square) contrast normalization. The RMS contrast is the most used normalization. It corresponds to the standard deviation of the luminance values and has been evidenced to be the most reliable indicator of the visibility of broadband filtered images [65]. In this control experiment, scenes were normalized to obtain a RMS contrast of 0.1 (i.e. 25.6 on a gray-level scale). This contrast normalization reduces contrast in LSF while enhancing HSF contrast. Results showed that elderly participants responded less often and made more false categorization when categorizing HSF than NF and LSF scenes even when contrast between spatial frequencies was equalized, whereas no differences were observed between spatial frequencies in young participants. They also responded less often and made more false categorization than young participants only for categorizing HSF scenes. Results revealed also that categorization of HSF scenes by elderly participants was improved by the RMS contrast normalization. However, it should be noted that RMS contrast normalization induces severe modifications in the amplitude spectrum properties of scenes that may bias behavioral and neurobiological responses.

## Brain Activation Results

### Whole Brain Analysis

The interaction between Groups (young and elderly participants) and Spatial frequencies (LSF, HSF and NF) was associated with isolated activation in the right inferior occipital gyrus (peak coordinate: 20x, -82y, -7z, F = 19.51). The nature of this interaction was explored firstly by calculating the contrasts that would allow us to compare the processing of different spatial frequencies for each group (within-group analysis), and then the contrasts that directly compared groups for each spatial frequency content (between-group analysis).

#### Within-group analysis

**Fig 3:**
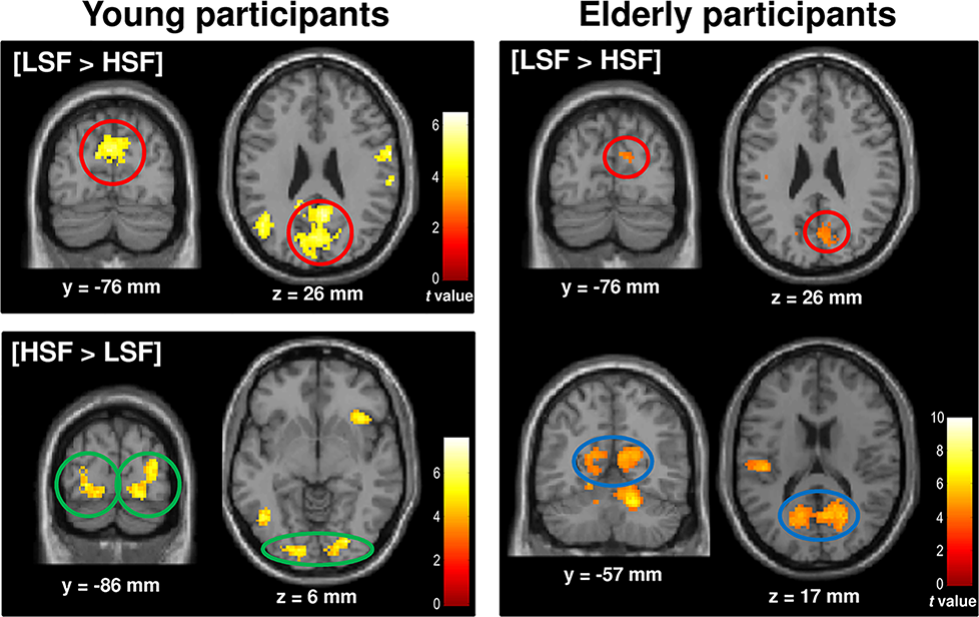
Cerebral regions activated by contrasting natural scenes filtered in LSF and HSF to fixation periods, and to each other in young and elderly participants. A retinotopic organization for spatial frequency processing was observed in young participants: medial activation of the anterior part of the cuneus for LSF (red circles), extending to the retrosplenial cortex (blue circles), and more lateral activation of the posterior part of the cuneus for HFS (green circles). In elderly participants, LSF scene categorization elicited bilateral activation of the retrosplenial (blue circles) cortex, extending to the anterior part of the cuneus (red circles). For illustrative purposes, statistical maps were generated with P < .0001 uncorrected.

Results for the within-group analysis are shown in Table 2 and Fig 3. We began by contrasting the processing of natural scenes filtered in LSF to HSF ([LSF > HSF] contrast) by young participants, and observed extensive bilateral recruitment of the occipital cortex (in the cuneus), the posterior cingulate gyrus (involving the retrosplenial cortex in the right hemisphere), the left middle temporal gyrus, the left superior and inferior parietal lobule areas, and the right postcentral gyrus. The opposite contrast ([HSF > LSF] contrast) showed that HSF scenes activated the occipital cortex bilaterally and the left inferior temporal gyrus. Critically, in the occipital cortex, results showed that LSF processing specifically activated the medial aspect of the occipital lobe, in the anterior half of the calcarine fissures (peak coordinates: 0x, -69y, 27z; Fig 3 and Table 2A). The reverse [HSF > LSF] contrast elicited significant, rather more posterior bilateral activation in the cuneus (right hemisphere: 20x, -82y, -7z; left hemisphere: -22x, -89y, 4z; Fig 3 and Table 2A). It should be noted that in the right hemisphere, the activation overlapped with the one associated with the interaction between groups and spatial frequencies. Contrasts relative to NF scenes revealed no significant activation.

**Table 2:** Cerebral regions specifically activated during the categorization of scenes for (a) young and (b) elderly participants. The statistical significance threshold for individual voxels was set at P < 0.05 FWE corrected for multiple comparisons (T > 5.18). Only the contrasts revealing significant activation are reported. For each cluster, the region showing the maximum T value is listed first, followed by the other regions belonging to the cluster [between brackets]. The Talairach coordinates (x, y, z) of the peak and the spatial extent (k = number of voxels in the cluster) are indicated.

Contrast	Area	Side	BA	k	x	y	z	T
**(a) Young participants**								
**[LSF > HSF]**	Cuneus	L/R	17/18	11	0	-73	30	5.49
	Posterior cingulate gyrus	L	31	27	-17	-37	34	6.45
		R	31	13	5	-49	26	5.87
	Middle temporal gyrus	L	39	13	-44	-68	36	6.00
	Superior parietal lobule	L	7	17	-24	-33	62	6.15
	Inferior parietal lobule/Supramarginal gyrus	L	40	6	-59	-28	23	5.86
	Postcentral gyrus	R	1/2/3	65	60	-1	13	6.50
**[HSF > LSF]**	Inferior occipital gyrus	R	18	20	20	-82	-7	6.51
	Middle occipital gyrus	R	19	7	33	-81	17	5.59
	Cuneus	L	17	6	-14	-87	-2	5.50
	Inferior temporal gyrus	L	37	10	-44	-58	-3	5.76
	Middle frontal gyrus	R	9	84	43	38	22	7.57
	Insula	R	-	27	38	22	-4	5.91
**[HSF-6 > HFS-3]**	Middle frontal gyrus	R	9	17	45	35	19	6.13
	Inferior frontal gyrus/Insula	R	47	16	33	22	-2	5.68
**[HSF-12 > HFS-3]**	Inferior frontal gyrus/Insula	R	47	123	35	25	1	6.92
	Middle frontal gyrus	R	9	27	45	35	19	6.59
	Anterior cingulate gyrus	L/R	32	19	-7	24	37	5.96
**(b) Elderly participants**								
**[LSF > HSF]**	Posterior cingulate gyrus	R	31	42	10	-59	16	5.87
	[Cuneus]	R	19		8	-71	28	5.57
	Posterior cingulate gyrus	L	31	6	-17	-62	16	5.50
	Superior temporal gyrus	L	22	5	-47	-18	19	5.56
	Postcentral gyrus	L	1/2/3	498	-42	-28	59	8.52
	[Precentral gyrus]		4		-39	-11	58	8.15
	Medial frontal gyrus/SMA	L	6	30	-22	-4	66	5.95
		R	6	18	18	-4	57	6.71
	Anterior cingulate gyrus	L/R	32	13	5	3	43	6.15
	Cerebellum	R	-	125	15	-51	-16	9.93

Abbreviations: R = right hemisphere; L = left hemisphere; BA = Brodmann area.

The [LSF > HSF] contrast for elderly participants showed that LSF processing specifically activated the posterior cingulate gyri bilaterally (in the retrosplenial cortex, extending to the anterior part of the cuneus) and the left superior temporal gyrus. Greater activation in the left motor area, the right cerebellum, and the anterior cingulate gyrus was also observed for the categorization of LSF than for HSF scenes. The opposite contrast [HSF > LSF] contrast revealed no significant activation considering the statistical threshold used in the present study (p < 0.05 FWE corrected for multiple comparisons. It should be noted that we observed an extensive recruitment of the right middle frontal gyrus (peak coordinates: 43x, 38y, 22z) for the processing of HSF at a more lenient threshold (p < 0.001 uncorrected). Once again contrasts relative to NF scenes revealed no significant activation. However, similarly to the [LSF > HSF] contrast, the [NF > HSF] contrast revealed a bilateral activation of the posterior cingulate gyri (BA 31, peak coordinates: 3x, -57y, 27z) extending to the anterior part of the cuneus at a more lenient threshold of p < 0.001 uncorrected. This result is not surprising given that in NF scenes, the whole frequency spectrum was present (LSF and HSF information). Therefore, the [NF > HSF] contrast in which activation from HSF scenes was subtracted from NF scenes indirectly highlight activation related to LSF processing.

#### Between-group analysis

**Fig 4:**
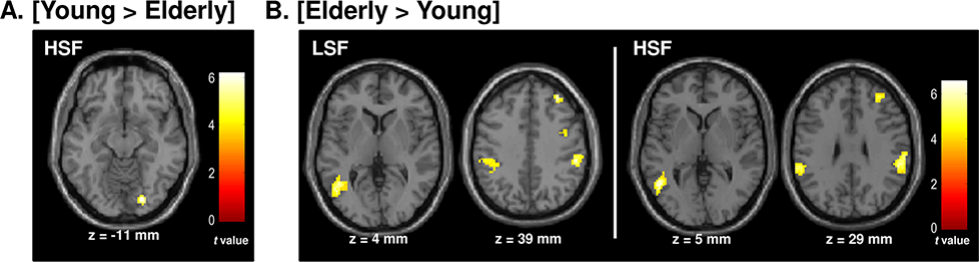
Cerebral regions differentially activated by (a) young and (b) elderly participants during the categorization of low-spatial frequency (LSF) and high-spatial frequency (HSF) scenes. For illustrative purposes, statistical maps were generated with P < .0001 uncorrected.

Results for the between-group analysis are shown in Table 3 and Fig 4. The between group analysis revealed greater activation of the right inferior occipital gyrus (peak coordinates: 20x, -82y, -7z) in young participants ([Young > Elderly] contrast) for the categorization of HSF scenes. This activation overlapped with the one associated with the interaction between groups and spatial frequencies. No greater activation was observed for the categorization of LSF and NF scenes. In contrast, greater activation in the left temporal areas (the middle temporal gyrus) and parietal areas bilaterally (the inferior parietal lobules), as well as in the right superior frontal gyrus was observed in elderly participants ([Elderly > Young] contrast) for the categorization of both LSF and HSF scenes. A similar cerebral network was activated during the categorization of HSF, with the exception of the middle temporal gyrus which showed greater activation only in the left hemisphere, the primary motor cortex where activation was greater only in the right hemisphere and the putamen and cerebellum where no significant activation appeared. No greater activation was observed for the categorization of NF scenes.

**Table 3:** Brain activity comparison between young and elderly participants for spatial frequency processing (LSF and HSF). The statistical significance threshold for individual voxels was set at P < 0.05 FWE corrected for multiple comparisons (T > 5.23). The Talairach coordinates (x, y, z) of the peak and the spatial extent (k = number of voxels in the cluster) are indicated.

	LSF	HSF
	x, y, z	x, y, z
**[Young > Elderly]**		
Right inferior occipital gyrus (BA 18)	-	20, -82, -7 (12)
**[Elderly > Young]**		
Left middle temporal gyrus (BA 37)	-49, -60, 5 (38)	-51, -58, 5 (31)
Left inferior parietal lobule (BA 40)	-51, -29, 36 (6)	-54, -35, 26 (52)
Right inferior parietal lobule/supramarginal gyrus (BA 40)	55, -29, 36 (7)	57, -32, 28 (106)
Right middle frontal gyrus (BA 9)	33, 41, 33 (8)	33, 43, 27 (27)

Abbreviations: BA = Brodmann area.

#### Effect of spatial frequency cut-off

The interaction between Groups (young and elderly participants) and LSF cut-offs (0.5, 1, and 2 cpd) was not significant. The interaction between Groups (young and elderly participants) and HSF cut-offs (3, 6, and 12 cpd) was associated with isolated activation in the anterior cingulate gyrus (peak coordinate: 0x, 28y, 25z, F = 18.80). We calculated the contrasts which enabled us to investigate the effect of the spatial frequency cut-off for each group (within-group analysis), and then the contrasts that compared groups directly for each spatial frequency cut-off (between-group analysis) in order to test the main effect of the interaction. The within-group analysis showed significant results only for young participants (Table 1). Activation in the cingulate gyrus was significantly greater for 12 cpd than for 3 cpd scenes ([HSF-12cpd > HSF-3cpd] contrast; 0x, 28y, 25z, F = 18.80). Furthermore, activation in the right inferior frontal gyrus (BA 47) and right middle frontal gyrus (BA 9) was significantly greater for both [HSF-12cpd > HSF-3cpd] and [HSF-6cpd > HSF-3cpd] contrasts. The between-group analysis revealed no significant results.

### ROI analysis

**Fig 5:**
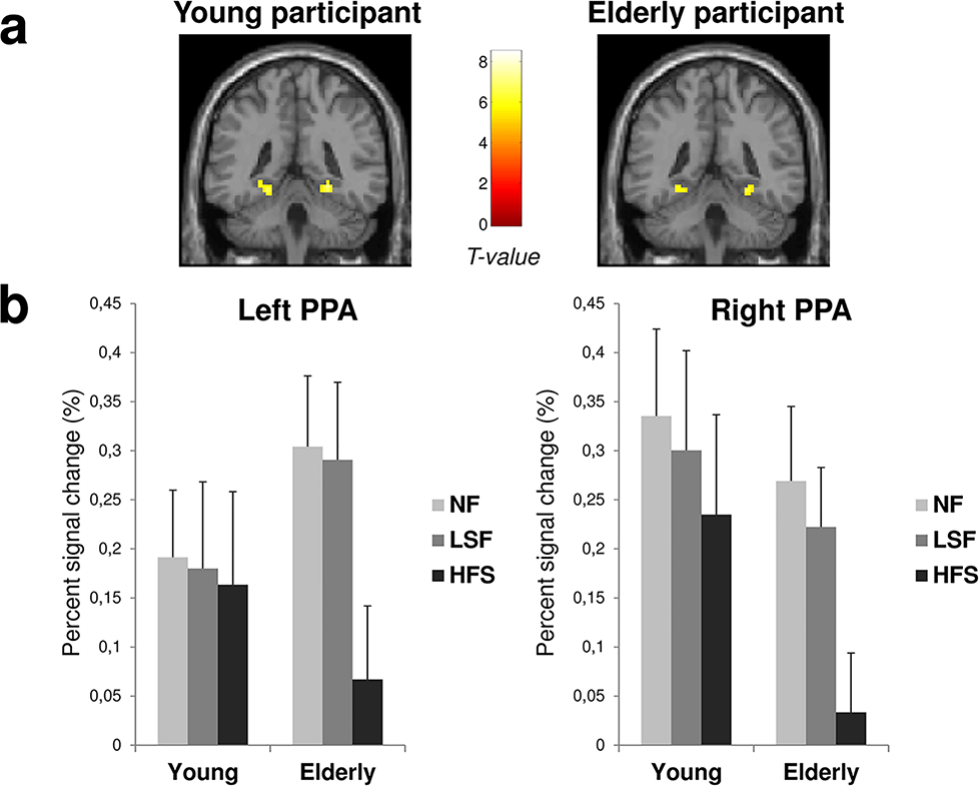
(a) Left and right parahippocampal place area (PPA) activated during the perception of scenes compared to faces and objects ([Scenes > Faces + Objects] contrast) for a young participant and an elderly participant. The ROIs were defined independently for each participant by contrasting scenes to other stimuli: [Scenes > Faces + Objects]. (b) Signal changes were then extracted from the scene-selective ROIs for each participant and each experimental condition (NF, LSF, and HSF). Graphics represent the mean percentage of signal change of the 11 young and 12 elderly participants for each ROI. Error bars correspond to the standard errors.

The PPA ROIs were defined in each individual, based on the independent Localizer experiment. This served as the structural constraint for the analysis of the data in the Spatial Frequency experiment. No recordings were obtained in one young participant due to technical problems. The PPA was localized for all other participants (11 young and 12 elderly participants) in the two hemispheres (see Fig 5A for an illustration on representative young and elderly participants) based on the ([Scenes > Faces + Objects] contrast). Parameter estimates (% signal change relative to the global mean intensity of signal) of block responses were extracted from the two sphere ROIs for each participant. The average parameter of activity was calculated for each experimental condition. These values were submitted to a first ANOVA for mixed designs with Groups (young and aged participants) as between-subject factor and Hemisphere (Left and Right) and Spatial frequency (NF, LSF, and HSF) as within-subject factors. The three spatial frequency cut-offs used for LSF and HSF scenes were grouped together for this ANOVA. There was no main effect of Groups (F_1,21_ < 1) but there was a main effect of Spatial frequencies (F_2,42_ = 15.83, p < 0.05) and a significant Spatial Frequency × Group interaction (F_2,42_ = 5.86, p < 0.05; Fig 5B). Planned comparisons showed that for young participants, there was no effect of Spatial frequencies (NF vs. HSF: F_1,21_ = 1.86, p = 0.19; LSF vs. HSF: F_1,21_ < 1; NF vs. LSF: F_1,21_ < 1), whereas for elderly participants, HSF scenes elicited less activation than did NF and LSF scenes (NF vs. HSF: F_1,21_ = 27.52, p < 0.05; LSF vs. HSF: F_1,21_ = 26.06, p < 0.05; NF vs. LSF: F_1,21_ < 1). Hemispheres did not interact with either Groups (F_1,21_ = 1.42, p = 0.25), Spatial frequencies (F_2,42_ = 1.85, p = 0.17), or their interaction (F_2,42_ = 2.77, p = 0.07).

Then, we tested whether the spatial frequency cut-off for the LSF and HSF scenes could influence the activation observed within PPA. Separate ANOVAs for mixed designs were conducted for the LSF and HSF stimuli with Groups (young and aged participants) as between-subject factor, and Hemisphere (Left and Right) and Spatial frequency cut-off (either 0.5, 1, and 2 cpd for LSF scenes or 3, 6, and 12 cpd for HSF scenes) as within-subject factors. For LSF, there was not effect of spatial frequency cut-off neither for young participants (0.5 vs. 1 cpd: F_1,21_ = 2.33, p = 0.14; 0.5 vs. 2 cpd: F_1,21_ < 1; 1 vs. 2 cpd: F_1,21_ = 1.68, p = 0.21) nor for elderly participants (all F_1,21_ < 1). For HSF, there was not effect of spatial frequency cut-off for young participants (all F_1,21_ < 1), whereas HSF-6cpd and HSF-12cpd scenes elicited less activation than HSF-3cpd scenes for elderly participants (3 vs. 6 cpd: F_1,21_ = 5.74, p < 0.05; 3 vs. 12 cpd: F_1,21_ = 13.98, p < 0.05; 6 vs. 12 cpd: F_1,21_ = 2.62, p = 0.12).

## Discussion

The present fMRI study investigated for the first time the effects of age-related differences on spatial frequency processing during the categorization of visual scenes. Overall, our results suggest that differences exist in spatial frequency processing in young and elderly participants, and are characterized by a deficit in low-contrasted HSF processing in older participants compared to younger participants.

### Behavioral HSF deficit in normal elderly people

Results showed that on a behavioral level, elderly participants gave more non-responses when categorizing HSF than NF and LSF scenes, irrespective of scene category, whereas no difference was observed between spatial frequencies in young participants. Elderly participants also made more non-responses than young participants during the categorization of HSF scenes. Complementary analyses revealed that as the spatial frequency cut-off of HSF scenes increased, elderly participants made more non-responses whereas no effect of cut-off was observed in young participants. These results suggest the presence of a selective impairment in elderly participants when processing HSF information. Musel et al. [45] previously investigated the categorization of filtered scenes in healthy elderly participants in order to establish normative data to assess the processing of spatial frequencies in age-related macular degeneration. They did not observe any selective HSF processing impairment due to age-related differences. However, their paradigm was constructed in order to avoid any bias in spatial frequency processing for healthy elderly controls. Presentation time, for example, was longer (300 ms vs. 100 ms in the present study) favoring HSF processing [10], and the spatial frequency cut-off of HSF scenes was very low (1 cpd vs. 3, 6, and 12 cpd in the present study) including thus middle spatial frequencies. Our complementary analyses conducted on spatial frequency cut-offs revealed that elderly participants are in fact sensitive to the amount of HSF information in a scene, and that performances on HSF processing are improved by a decrease in the cut-off value.

Concerning rapidity of categorization, elderly participants categorized scenes less rapidly than young participants. More specifically, their performance was poorer than that of younger participants when categorizing scenes filtered in HSF, in support to the impairment of HSF processing observed in response accuracy in elderly participants. Furthermore, both young and elderly participants categorized LSF scenes more rapidly than HSF scenes, in favor of a temporal processing precedence of LSF over HSF. For young participants, this result is consistent with previous behavioral studies which used either hierarchal stimuli and showed a global precedence effect [35] or filtered scenes as stimuli and showed temporal precedence of LSF over HSF [8,9,10]. However, for elderly participants, this result is not consistent with previous studies conducted in normal elderly people which used hierarchical forms as stimuli [39,41]. These studies showed either a local precedence effect or a reduced global precedence effect in older participants, and suggest the existence of a selective impairment in the processing of LSF, which convey global information, associated with normal aging. However, it should be noted that the relationship between spatial frequencies and local and global information in hierarchical stimuli is far from univocal [66]. Global information can, for example, be conveyed by not only LSF but also by HSF. When attempting to understand spatial frequency processing, any inferences based on hierarchical stimuli should therefore be considered with caution. Our results were also not consistent with previous behavioral studies which used filtered scenes as stimuli. Using sequences of filtered scenes simulating either a LSF to HSF processing or a reverse HSF to LSF processing as stimuli, Musel et al. [8] showed that elderly participants categorized outdoor scenes faster when LSF were presented before HSF, but they categorized indoor scenes faster when HSF was presented before LSF information. These results suggest a either LSF or HSF temporal precedence in normal elderly people depending on the perceptual properties of categories, which is not consistent with the LSF temporal precedence observed irrespective of categories in the present study.

The discrepancies between our results and previous findings on hierarchical stimuli and scenes could be explained by the difficulty of the visual task. Critically, in all the studies mentioned above, error rates were very low even for elderly participants. This suggests that the tasks used were not suited to the investigation of visual deficits, but rather to the investigation of the temporal aspects of the visual processing. Interestingly, results showed that elderly participants were always slower than young participants. Musel et al. [8] previously hypothesized that elderly participants privileged high accuracy to the detriment of speed using additional processes that lengthened reaction times. In fact, they would process further semantic information to categorize scenes. Such additional processes would have changed the temporal precedence of spatial frequency processing depending on the categories. For example, they would preferentially use global spatial invariants in LSF (such as the ground, the sky, and the direction of natural light) for outdoor categorization, leading to a temporal precedence of LSF over HSF processing, and local elements in HSF (e.g., table, sofa, chair) for indoor categorization, leading to a temporal precedence of HSF over LSF processing. The preferred HSF information for categorizing indoor scenes in elderly people may explained why, in the present study, the HSF deficit in elderly participants was more pronounced for indoor than outdoor scenes. Indeed, elderly participants responded less often for categorizing indoors than outdoors only when scenes were filtered in HSF. This result suggests that the HSF deficit increased when the categorization task is preferentially performed on the basis of HSF information.

To summarize, our behavioral results point to a selective deficit in the categorization of HSF scenes in elderly people, which is consistent with the loss of contrast sensitivity to HSF previously observed in studies using sinusoidal gratings [26,27,29,30]. It should be noted that the results from the control behavioral experiment, in which the luminance contrast between NF, LSF and HSF scenes was equalized, revealed that performance of elderly participants was than that of younger participants when categorizing scenes in HSF. However, their performance was improved by the enhanced contrast in HSF scenes, suggesting that the visual deficit of scene perception in elderly participants was also partially driven by the low luminance contrast in HSF scenes. Further research is needed to clarify the extent to which luminance contrast influences spatial frequency processing during scene perception in elderly people. For example, this could be done by systematically manipulating different levels of luminance contrast as a function of different spatial frequencies.

### Neurobiological correlates of the HSF deficit

The neural correlates of spatial frequency processing were investigated under fMRI. We compared activation elicited by LSF and HSF scenes in young and elderly participants separately (within-group analysis), and then we compared groups for each type of spatial frequency content (between-group analysis). The within-group analysis revealed a retinotopic organization of spatial frequency processing in young participants. The categorization of LSF scenes (relative to HSF) activated the medial aspect of the occipital lobe in the anterior half of calcarine fissure linked to the peripheral visual fields, in accordance with the retinotopic properties of early visual areas. The categorization of HSF scenes (compared to LSF) elicited activation in the posterior part of the occipital lobes, which are linked to the fovea. Similar results were observed previously in fMRI studies conducted on young adults using either sinusoidal gratings [12,14] or filtered scenes [13]. Retinotopic processing of spatial frequencies could be explained by the distribution and neurophysiological properties of photoreceptor and ganglion cells in the human retina [67,68]. The density of cones and midget ganglion cells, which are used to process HSF information, is greatest in the fovea. Because the fovea is represented in the posterior parts of the visual areas, HSF information may be predominantly processed in these areas. In contrast, the density of rods and parasol ganglion cells, which are used to process LSF information, increases with foveal eccentricity. Because the peripheral retina is represented in progressively more anterior parts of the visual areas, LSF information may be predominantly processed in these areas.

However, retinotopic processing of spatial frequency was not always observed in elderly participants. LSF scene categorization elicited greater activation in the anterior part of the cuneus than HSF scene categorization. However, HSF scene categorization did not elicit stronger significant activation than LSF scenes. The absence of significant activation was consistent with the observed behavioral deficit in HSF processing in older participants compared to younger participants. This may result from anatomical and functional changes in the primary visual cortex associated with normal aging. Salat et al. [47], for example, observed a global thinning of the cerebral cortex, including the primary visual areas, with age. Brewer and Barton [48,49] showed that the size of the receptive field corresponding to central vision was larger in elderly than in young adults. To be precise, the size of the receptive fields corresponding to an eccentricity of 3° in elderly adults (over the age of 57) is equivalent to one of 8° in young adults (under the age of 36). This result may suggest that absence of the receptive field tuned to HSF processing in elderly people. The authors also showed that the surface of projection of the fovea was smaller in the elderly than in young adults [49], suggesting a decrease in the cortical surface dedicated to HSF processing. Further anatomical studies using, for instance, voxel-based morphometry (VBM) should be conducted to determine whether the HSF deficit in normal elderly participants could be linked to changes in gray matter in the visual areas related to central vision.

Direct comparison between groups (between-group analysis) revealed greater activation of the right inferior occipital gyrus in young participants (peak coordinates: 20x, -82y, -7z) than in elderly participants for the categorization of HSF scenes only (greater activation was not observed for NF and LSF scenes). This activation overlaps with the retinotopic processing of HSF information in the right hemisphere in young participants (peak coordinates: 20x, -82y, -7z), and mirrors the absence of HSF specific significant activation in older participants (within-group analysis). Overall, results from within- and between-analyses suggest that an occipital cortex dysfunction underlies HSF scene categorization in elderly people. However, a high level of activation was noted in a temporo-parietal cortical network in elderly participants for the categorization of LSF and HSF (but not NF) scenes. We cannot rule out that this enhanced activity was linked to increased attentional resources in elderly participants.

Firstly, we observed greater activation in the inferior parietal lobules, with a greater extent for HSF than LSF scenes. These regions have previously been described as mediating and sustaining the allocation of attention to low and high spatial frequencies in scenes [69,70] and may be engaged by elderly participants to emphasize spatial frequency information, in particular HSF. We also observed stronger activation in the right middle frontal gyrus. It should be noted that, for elderly participants, only the right middle frontal gyrus was more activated by the processing of HSF than LSF at a more lenient statistical threshold. This region has previously been described as being involved in sustained attention [71] and in people with low vision when more attentional resources are required for words recognition [72]. Both activations therefore suggest that for elderly people the categorization of filtered scenes requires greater attentional resources.

We also observed stronger activation in the left posterior middle temporal cortex in elderly participants during the categorization of both LSF and HSF scenes. This region is known to be involved in the semantic categorization of visual stimuli [70,73,74,75], and could be linked to the retrieval of semantic concepts related to the visual stimulus [74]. Alternatively, this region is also known to be involved in word retrieval [76] and word generation [77]. Its activation could be related to internal dialogue which elderly participants used to generate the semantic category label (indoor or outdoor). High-level visual areas associated with semantic categorization were, therefore, more highly activated in older than in young participants. The elderly participants may engage such additional semantic processes to compensate for their deficit in low-level vision and diminished occipital cortex activation. Interestingly, despite their behavioral deficit when categorizing HSF scenes, persistent activation in the left posterior middle temporal cortex was present in this experimental condition. Stronger activation of left high-level visual areas (left middle temporal gyrus) paralleled with weaker activation of right low-level visual areas (right inferior occipital gyrus). It seems that elderly participants made more demands on areas in the left than in the right hemisphere in order to categorize HSF scenes. This is consistent with the involvement of verbal processes for which the left hemisphere is dominant. However, it should be noted that a greater activation of the left temporal cortex, but also of the left primary motor cortex (BA 4) and right cerebellum directly linked to the motor response given by participants, was observed for LSF than HSF scenes for elderly participants (see the within-group analysis), and is consistent with the higher non-response rate observed for the categorization of HSF scenes.

Finally, we also investigated age-related differences in spatial frequency processing in the PPA. The PPA was evidenced to be a scene-selective cortical region [53,55]. This region has been linked to high-order functions during scene perception, such as navigation, spatial layout processing and scene recognition [54,56,78,79,80], but also contextual association [52,81]. Recent studies revealed that this region is also sensitive to spatial frequency information in scenes [59,60,82]. In a recent fMRI study in which the experimental paradigm was the same as that used in the present study [59], results showed that NF and LSF scenes elicited greater activation than HSF scenes in the PPA for young participants. The present study showed that activation of the PPA did not differ between spatial frequencies for young participants maybe due to the smaller sample size (12 instead of 16 young participants). Importantly, results showed that the age of participants interacted with the processing of spatial frequencies in the PPA. Activation of the PPA significantly decreased during HSF scene categorization (compared to LSF) for elderly participant, suggesting that the specific age-related HSF deficit could also impact the processing of spatial frequencies in PPA.

## Conclusion

The present findings indicate a specific low-contrasted HSF deficit in elderly people, in association with occipito-temporal cortex dysfunction. Our study provides important additional information for the development of models of scene perception and has an impact on public health. Understanding of the cerebral interpretation of scenes in elderly people is therefore of primary importance for the improvement of their quality of life. Our study provides also further support to the understanding of visual impairments in pathological aging. Indeed, the number of visual impairments related to age is in constant progression in developed countries. Age-related macular degeneration (AMD) is the first cause of central vision loss in the elderly population and it mainly affects people over the age of 50 [83,84,85]. The investigation of spatial frequency processing in natural environments is even more important since elderly people with AMD exhibit a specific deficit in the processing of HSF in photographs of natural scenes [45].

## Supplementary Material

A control behavioral study was conducted in the same participants (12 young and 12 elderly participants), in which the luminance contrast of filtered stimuli was equalized using a RMS (root mean square) contrast normalization. Scenes were normalized to obtain an RMS contrast of 0.1 (i.e. 25.6 on a gray-level scale). We chose a value situated between LSF and HSF contrast values in natural conditions (i.e. 0.21 and 0.04, respectively in the LUM condition), in order to avoid affecting one spatial frequency condition more than another. This contrast normalization reduces contrast in LSF while enhancing HSF contrast. Participants performed the same categorization task except that scenes with equalized RMS contrast replaced the original scenes.

Results showed that the global performance was improved by the RMS contrast normalization (mNR: 1.9 ± 3.3%, mFC: 4.1 ± 5.9%, mRT: 632 ± 83 ms) in comparison with the original experiment (mNR: 9.7 ± 7.2%, mFC: 5.6 ± 7.3%, mRT: 657 ± 87 ms). However, results still showed that elderly participant performance was poorer than that of younger participants when categorizing scenes filtered in HSF only. Three 2x2x2 analyses of variance (ANOVA) with Groups (young and elderly participants) as between-subjects factor, and Spatial frequencies (NF, LSF, and HSF) and Categories (indoors and outdoors) as within-subjects factors were conducted on mNR, mFC and mRT. The ANOVA on mNR revealed that elderly participants responded less often than young participants (3.1 ± 4.9% and 0. 1 ± 0.1%, respectively; F_1,22_ = 4.30, p < 0.05). The expected Group x Spatial frequency interaction was significant (F_2,44_ = 4.42, p < 0.05). Planned comparison showed that elderly participants responded less often when categorizing HSF scenes (6.1 ± 9.2%) than NF scenes (0.1 ± 0.2%; F_1,22_ = 7.49, p < 0.05) and LSF scenes (2.2 ± 3.8%; F_1,22_ = 7.32, p < 0.05), while there was no difference between LSF and NF scenes (F_1,22_ = 2.17, p = 0.15). For young participants, there was no effect of spatial frequencies (NF: 0.1 ± 0.2%; LSF: 0.1 ± 0.2%; HSF: 0.1 ± 0.1%; all F_1,22_ < 1). In addition, elderly participants responded less often than young participants only when categorizing HSF scenes (F_1,22_ = 5.35, p < 0.05; NF: F_1,22_ < 1; LSF; F_1,22_ = 1.12, p = 0.30). The ANOVA on mFC did not show a main effect of Groups (F_1,22_ < 1). The Group x Spatial frequency interaction was significant (F_2,44_ = 6.84, p < 0.01). Planned comparison showed that elderly participants made more false categorizations when categorizing HSF scenes (7.0 ± 8.0%) than NF scenes (0.3 ± 0.7%; F_1,22_ = 7.43, p < 0.05) and LSF scenes (4.9 ± 6.9%; F_1,22_ = 14.86, p < 0.001), and when categorizing LSF than NF scenes (F_1,22_ = 5.52, p < 0.05). For young participants, there was no effect of spatial frequencies (NF: 3.3 ± 4.3%; LSF: 3.4 ± 5.0%; HSF: 2.8 ± 4.1%; all F_1,22_ < 1). Finally, the ANOVA on mRT revealed a main effect of Groups (F_1,22_ = 5.95, p < 0.05). Elderly participants were slower to categorize scenes than healthy participants (671 ± 82 and 593 ± 82 ms, respectively). The Group x Spatial frequency interaction was not significant (F_2,44_ = 2.18, p = 0.12). There was a main effect of Spatial frequency (F_2,44_ = 23.10, p < 0.001). All participants had longer RT when categorizing HSF scenes (661 ± 91 ms) than NF scenes (601 ± 76 ms; F_1,22_ = 37.30, p < 0.001) and LSF scenes (633 ± 82 ms; F_1,22_ = 10.69, p < 0.005), and when categorizing LSF than NF scenes (F_1,22_ = 15.81, p < 0.001).
